# The Impact of Nutrition Education Based on the PRECEDE‐PROCEED Model on Improving the Nutritional Status and Growth Status of 7–12‐Year‐Old Malnourished Children in Kermanshah

**DOI:** 10.1002/fsn3.70879

**Published:** 2025-08-28

**Authors:** Fatemeh Maleki, Shaghayegh Sharifi Soltani, Shahab Rezaeian, Mahmood Ghasemi, Mehnoosh Samadi

**Affiliations:** ^1^ Student Research Committee, School of Nutritional Sciences and Food Technology Kermanshah University of Medical Sciences Kermanshah Iran; ^2^ Department of Nutritional Sciences, School of Nutritional Sciences and Food Technology Kermanshah University of Medical Sciences Kermanshah Iran; ^3^ Infectious Diseases Research Center Kermanshah University of Medical Sciences, Research Institute for Health Kermanshah Iran; ^4^ Department of Pediatrics, School of Medicine Kermanshah University of Medical Sciences Kermanshah Iran

**Keywords:** precede‐proceed model, stunting, underweight, wasting

## Abstract

Malnutrition significantly affects children's health and social well‐being, leading to growth disorders, such as underweight and short stature. This study investigated the impact of nutrition education based on the PRECEDE‐PROCEED Model and the optimal food basket recommended by the Ministry of Health on the growth status and dietary intake of malnourished children aged 7 to 12 years. A controlled trial was conducted with 254 malnourished children in Kermanshah, randomly divided into intervention (127) and control groups. Data were collected at three intervals: before, 6, and 12 weeks after the educational intervention. The intervention consisted of eight educational sessions lasting 45–60 min each. Results showed that the nutrition education program significantly improved components of the PRECEDE model, including knowledge, attitude, self‐efficacy, and behavior (*p* < 0.001). The intervention group experienced significant increases in Weight for Age Percentile and BMI for Age Percentile (*p* < 0.001), though no significant difference was found in Height for Age Percentile (*p* = 0.929). Dietary intake data revealed significant increases in mean calories, protein, vitamin A, riboflavin, iron, and zinc (*p* < 0.001), whereas vitamin C and D intakes showed no significant differences (*p* = 0.675 and *p* = 0.610, respectively). In conclusion, nutrition education based on the PRECEDE model can effectively enhance dietary intake and reduce underweight and thinness in children by improving knowledge, attitude, and self‐efficacy.

## Graphical abstract

The current results found that nutrition education based on the PRECEDE‐PROCEED Model can increase awareness, attitude, self‐efficacy, and encourage parents and teachers to create positive nutritional behaviors as well as improve food intake in school‐age children, thereby reducing the incidence of underweight and wasting in them.

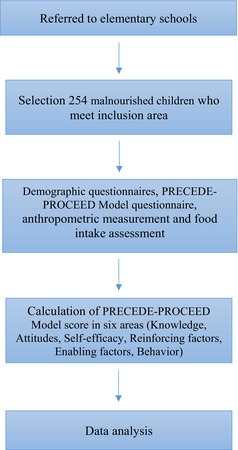

## 1 Introduction

Malnutrition is a common disease worldwide and is considered one of the public health concerns and causes of death in children (Bidira et al. 2022). The World Health Organization (WHO) defines the term “malnutrition” as a nutritional imbalance, including nutritional deficiency covering growth retardation, weight loss, short stature, and micronutrient deficiencies, as well as overnutrition, including overweight and obesity (World Health Organization 2020). Nutritional conditions are compromised more quickly during childhood than at other ages, so it is of utmost importance to take appropriate therapeutic measures to reduce the burden of malnutrition and its complications (Tadayyon et al. 2025). Children in developing countries and poor areas are more at risk of malnutrition complications. The WHO, the United Nations Children's Fund (UNICEF), and the World Bank Group estimated the prevalence of malnutrition in 2022 at 149 million children under 5 years of age who were stunted (short for age) and 45 million children who were underweight for height (World Health Organization 2020). The prevalence of malnutrition in Iran based on Weight for age percentile (WFA), Height for age percentile (HFA), and Weight for height percentile (WFH) is 19%, 20%, and 12.5%, respectively (Mohseni et al. 2022; Sinha et al. 2018). Linear growth is greater during childhood than at other times, and since school‐age children are more prone to poor nutritional behaviors, they are more at risk of malnutrition and its consequences, including developmental disorders, cognitive dysfunction, and physical diseases (Mohseni et al. 2022; Teo et al. 2019). Studies have shown that eating habits and patterns are formed in elementary school‐age, and enhancing parental awareness can establish a healthy lifestyle in children from the very beginning (Wahyuningsih et al. 2022). Therefore, it is possible to create healthy eating habits that lead to correct nutritional behaviors in adulthood at this age. Since one of the main reasons for malnutrition in children is insufficient health and nutrition information, nutritional education for children and parents, as well as increasing their knowledge and skills in this field, can lead to proper growth and development of children. Accordingly, this study was designed and implemented with the aim of examining the effect of nutritional education on improving the nutritional and growth status of primary school‐age children suffering from malnutrition in Kermanshah city (Charlton et al. 2021; Mitra et al. 2020).

## 2 Materials and Methods

This randomized controlled trial study was conducted on school‐age children (7–12 year old) in Kermanshah. Data were collected on three occasions (baseline, 6, and 12 weeks after the intervention), where demographic questionnaires (age, gender, parental education level, birth order, birth weight, household size, and monthly income) and the PRECEDE‐PROCEED Model questionnaire were used. PRECEDE is derived from the words Predisposing, Reinforcing, Enabling, Construct, Ecological/Educational, Diagnosis, and Evaluation. PROCEED is also a combination of the words Policy, Regulatory, Organizational, Construct, Educational, Environmental, and Development. A 3‐day food record questionnaire was employed to assess dietary intake, whereas Centers for Disease Control and Prevention (CDC) charts, which are classified based on percentiles, were utilized to assess growth (Kuczmarski et al. 2000, 2002; Yang et al. 2010). Height for age, weight for age, and body mass index for age are important indicators of nutritional status and determine stunting, underweight, and wasting in children. Stunting, underweight, and wasting are determined by a Z score of < −2, whereas severe underweight, severe short stature, and severe wasting are determined by a *Z* score of < −3 (Sinha et al. 2018). The questionnaires were completed by the parents of the students once the questions were explained to them by the trainer.

### 2.1 Procedure

**FIGURE 1:**
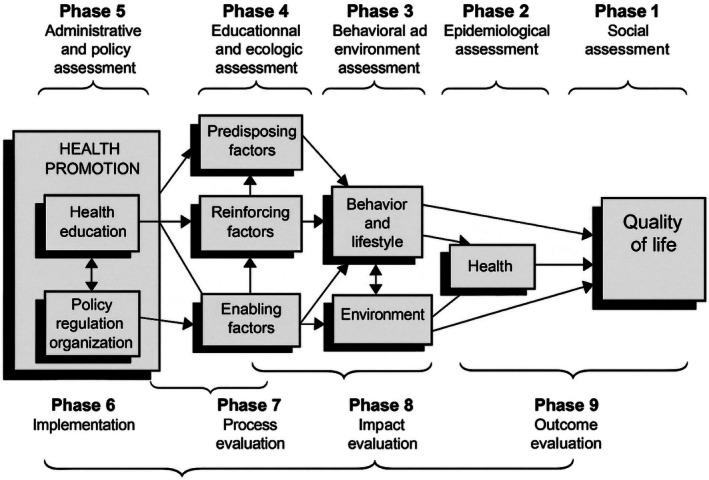
PRECEDE‐PROCEED model.

The intervention group was placed under the educational program based on the PRECEDE‐PROCEED Model, which is a planning model for identifying needs and changing behavior (Binkley and Johnson 2013). As displayed in Figure 1, this model includes nine stages in planning, implementing, and evaluating a health promotion program. Part of this model is PRECEDE, which was carried out with the aim of assessing educational needs, identifying goals, and determining priorities (stages 1–5). The PROCEED section includes the implementation stage, process evaluation, impact evaluation, and outcome evaluation (stages 6–9) (Pourhaji et al. 2020). Educational needs assessment as well as prioritization and determination of educational goals were carried out according to the initial PRECEDE questionnaire. The training provided involved teaching food groups, how to prepare foods according to the child's interests, healthy nutrition, food diversity, meat alternatives, protein sources, and foods containing vitamins and minerals such as vitamin A, riboflavin, iron, calcium, and zinc. In these trainings, first, sources containing micronutrients were introduced, then the desired micronutrients were introduced, and finally, the effect of consuming these sources and their micronutrients on child development was explained in simple language. The training was conducted weekly for 2 months, each session lasting 45–60 min, using face‐to‐face lectures, group discussions, worksheets, questions and answers, and educational videos, with the participation of students, parents, and teachers in school. In the control group, only anthropometric and dietary measurements were taken, whereas no intervention was performed.

### 2.2 PRECEDE‐PROCEED Model Details

PRECEDE‐PROCEED Model is a guide model for analyzing health problems and how to design an educational program with an emphasis on examining all factors affecting health status. This study will be conducted based on the Precede‐Proceed model, which consists of nine steps.

Step 1: is a social assessment that examines the quality of life of the community population and demographic information will be assessed to begin planning. Step 2: Epidemiological assessment, which is a complete description of the health problem and its occurrence in the study population, including mortality rates, prevalence, impact on individual functioning, and severity of disability. In this stage, we will examine prevalence and measure growth indicators (height for age, weight for age, weight for height, body mass index for age). Step 3: Behavioral and environmental assessment, which includes examining behavioral factors on health problems (malnutrition) such as unhealthy eating. In this section, the three‐day food diary method will be used to examine the nutritional status of children. Since our goal is not to influence environmental factors, only behavioral factors will be examined in this section. Step 4: Educational and ecological assessment, in which factors that can influence health behavior, including predisposing factors (Knowledge, attitude, and self‐efficacy), reinforcing factors (influence of others, family members, and peers), and enabling factors (availability of resources and skills), are examined and assessed using the PRECEDE questionnaire. Step 5: Administrative assessment and policy review, which includes assessing and measuring administrative, organizational, and resource facilities for developing and implementing a program and the limitations related to resources, policies, facilities, etc. that are being examined. In the present study, coordination with educators, school administrators, teachers, and parents, scheduling, budgeting, and coordinating the location and time of training sessions are carried out. Step 6: The implementation phase is when the intervention group will undergo a training program that, based on the information from the initial questionnaire, will first assess educational needs, prioritize these trainings, and determine educational goals. Then, these trainings will be conducted through face‐to‐face lectures, group discussions, worksheets, questions and answers, and educational videos with the presence of students, their parents, and teachers. Step 7: Process evaluation, which includes evaluating policies, resources, staff, service quality, and program implementation. In this study, for the process evaluation phase, research team members will review the implementation process, service quality, and behavior of the intervention group every week, and the pilot steering group will hold meetings to monitor data during the implementation phase. Step 8: Outcome evaluation, which includes evaluating the program's effects on intermediate objectives such as changing predisposing, enabling, reinforcing, and behavioral factors, will be conducted using questionnaires and statistical analyses. Step 9: Outcome evaluation, which includes evaluating the final and long‐term effects of the program and comparing it with the final goals such as changes in quality of life, health, and social indicators, will be conducted using questionnaires, measuring anthropometric indicators, and statistical analyses.

The PRECEDE questionnaire includes predisposing factors (knowledge, attitude, and self‐efficacy), reinforcing factors (the influence of others, family members, and peers), enabling factors (availability of resources and skills), and behavior assessment, which is assessed through a questionnaire. The predisposing factors questionnaire includes questions in the knowledge domain (about food groups, healthy eating, meat alternatives, protein sources, foods containing iron, calcium, and zinc, and the daily body requirement of food groups), attitude domain questions (feelings about eating meat, fruits, vegetables, and snacks), and self‐efficacy questions such as the ability to wake up early to eat breakfast or consume proteins such as eggs for breakfast or the ability to choose healthy snacks even when friends encourage them to eat unhealthy foods. The enabling factors questionnaire covers questions about available resources (such as nutrition education tools available to students and parents). The reinforcing factors questionnaire contains one question about encouraging teachers, parents, and peers to consume healthy foods (Ashourpour et al. 2017).

### 2.3 Dietary Intake Assessment

A 3‐day food record questionnaire (1 day off and 2 days off) was used to assess dietary intake, which was evaluated at baseline, Week 6, and Week 12 after the educational intervention. The intake of energy, macronutrients, and micronutrients, and water intake was measured using the Modified Nutritionist IV Software (version 3.5.2, First Data‐Bank; Hearst Corp., San Bruno, CA).

### 2.4 Anthropometric Assessment

Children's growth charts (Height for age Percentile (HFA), WFA, and BMI for age Percentile (BFA)) were assessed and interpreted at baseline, Week 6, and Week 12 of the educational intervention. The weight of the children was measured in a standing position, without shoes, and with minimal clothing using a digital scale with an accuracy of 100 g. Height was measured while standing, without shoes, using a nonelastic tape measure attached to the wall so that the back of the body and heels were attached to the wall and the child's gaze was straight ahead (Kamiab et al. 2020). Body mass index was also calculated by dividing weight by the square of height, after which, using growth charts, stunting, wasting, and underweight were diagnosed and compared to WHO reference for recommended cutoff values for the target population (Wijnhoven et al. 2013).

## 3 Statistical Analysis

Quantitative variables were described by mean and standard deviation, whereas qualitative variables were described by frequency and frequency percentage, as well as in terms of intervention and control groups. Quantitative variables were compared between the intervention and control groups by a *t*‐test, whereas qualitative variables were compared through a chi‐square test. In the analytical section, due to the interventional nature of the design, data were analyzed based on the Intention to treat method. One‐Way Repeated Measures ANOVA was employed to compare the mean outcomes before and after the study in each group, where the difference in between‐group means was examined with a 95% confidence interval. Linear regression was also used to determine the relationship between the intervention and outcomes (given the quantitative nature of the outcomes) and to adjust for the effect of confounding variables. All tests were two‐sided and the significance level was less than five hundredths. The collected data were analyzed using SPSS 26 software at a significance level of *p* < 0.05.

## 4 Results

### 4.1 Demographic Information

A total of 254 malnourished children participated in this study; nonparametric tests were rejected due to the validity of the assumptions. The demographic characteristics of 127 malnourished children in the intervention group and 127 malnourished children in the control group are reported in Table 1. As can be observed, the study groups were homogeneous and there was no significant difference in terms of demographic indicators between them (Table 1).

**TABLE 1:** Comparison of general characteristics of the studied individuals by the groups under investigation.

Variable		Group		*p*
		In tervention *N* (%)	Control *N* (%)	
Gender^a^	Girl	107 (84.3)	105 (82.7)	0.735
	Boy	20 (15.7)	22 (17.3)	
Birth rank^a^	First born	58 (45.7)	57 (44.9)	0.347
	Second born	55 (43.3)	43 (33.9)	
	Third born	12 (4.9)	23 (18.1)	
	Fourth born	2 (1.6)	4 (3.1)	
Family size^a^	Three people	29 (22.8)	39 (30.7)	0.91
	Four people	68 (53.5)	51 (40.2)	
	Five people	28 (22)	29 (22.8)	
	Six people	2 (1.6)	8 (6.3)	
Family income (Toman)^a^	< 5000,000	10 (7.9)	11 (8.7)	0.437
	5000,000–8000,000	52 (40.9)	41 (32.3)	
	8000,000–14,000,000	46 (36.2)	57 (44.9)	
	> 14,000,000	19 (15)	18 (14.2)	
Father education^a^	Under diploma	37 (29.1)	40 (31.5)	0.461
	Associate degree	42 (33.1)	48 (37.8)	
	Bachelor	44 (34.6)	21 (24.4)	
	Master	4 (3.1)	8 (6.3)	
Mother education^a^	Under diploma	44 (34.6)	39 (30.7)	0.478
	Associate degree	49 (38.6)	51 (40.2)	
	Bachelor	33 (26)	34 (26.8)	
	Master	1 (0.8)	3 (2.4)	
Birth weight (kg)^b^		3116 ± 333.6	3102 ± 316.5	0.348
Age (month)^b^		96.93 ± 16.81	97.27 ± 18.78	0.206
Weight (kg)^b^		19.69 ± 2.87	19.77 ± 3.33	0.132
Height (cm)^b^		121.22 ± 8.86	120.82 ± 9.54	0.342
BMI (kg/m^2^)^b^		13.35 ± 0.62	13.47 ± 0.62	0.798
Percentile^b^		3.140 ± 1.24	3.05 ± 1.27	0.775

^a^ Chi‐square.

^b^ *T* test.

### 4.2 Anthropometric Indicators

Table 2 outlines the findings related to anthropometric indicators at the beginning of the study and at 4 and 12 weeks after the intervention. As can be seen, significant changes were observed in the body weight of the children in the intervention group (WFA, *p* < 0.001). Also, a significant rise in body mass index was observed in the intervention group, such that the mean BFA increased from 3.47 at the beginning of the study to 5.05 and 6.98 at Weeks 4 and 12, respectively, post‐intervention (*p* < 0.001). In the present study, although there was an increase in the height growth of the children in the intervention group from Weeks 0 to 12, these changes were not statistically significant compared to the control group, and no change was observed in the children's HFA (*p* = 0.929) (Table 2).

**TABLE 2:** Mean anthropometric indices before and after intervention in the studied groups.

Variable	Intervention group				Control group				*p⃰*
	Week 0	Week 6	Week 12	*p*	Week 0	Week 6	Week 12	*p*	
Underweight Weight for age Percentile (WFA)	2.99 ± 1.26	4.24 ± 2.01	5.01 ± 2.60	< 0.001	2.89 ± 1.29	2.79 ± 1.39	2.73 ± 1.48	0.288	< 0.001
Stunting Height for age Percentile (HFA)	3.37 ± 1.09	3.41 ± 1.05	3.49 ± 1.11	0.218	3.42 ± 1.23	3.35 ± 1.23	3.41 ± 1.19	0.336	0.929
Wasting BMI for age Percentile (BFA)	3.47 ± 1.29	5.05 ± 1.69	6.98 ± 2.45	< 0.001	3.28 ± 1.16	3.53 ± 1.50	3.39 ± 1.33	0.714	< 0.001

*Note:* *p* values result from One‐Way Repeated Measures ANOVA test. Data are presented as mean ± SD (standard deviation), CI, confidence interval. *p* value is significant at *P* < 0.05.

### 4.3 PRECEDE‐PROCEED Model

**FIGURE 2:**
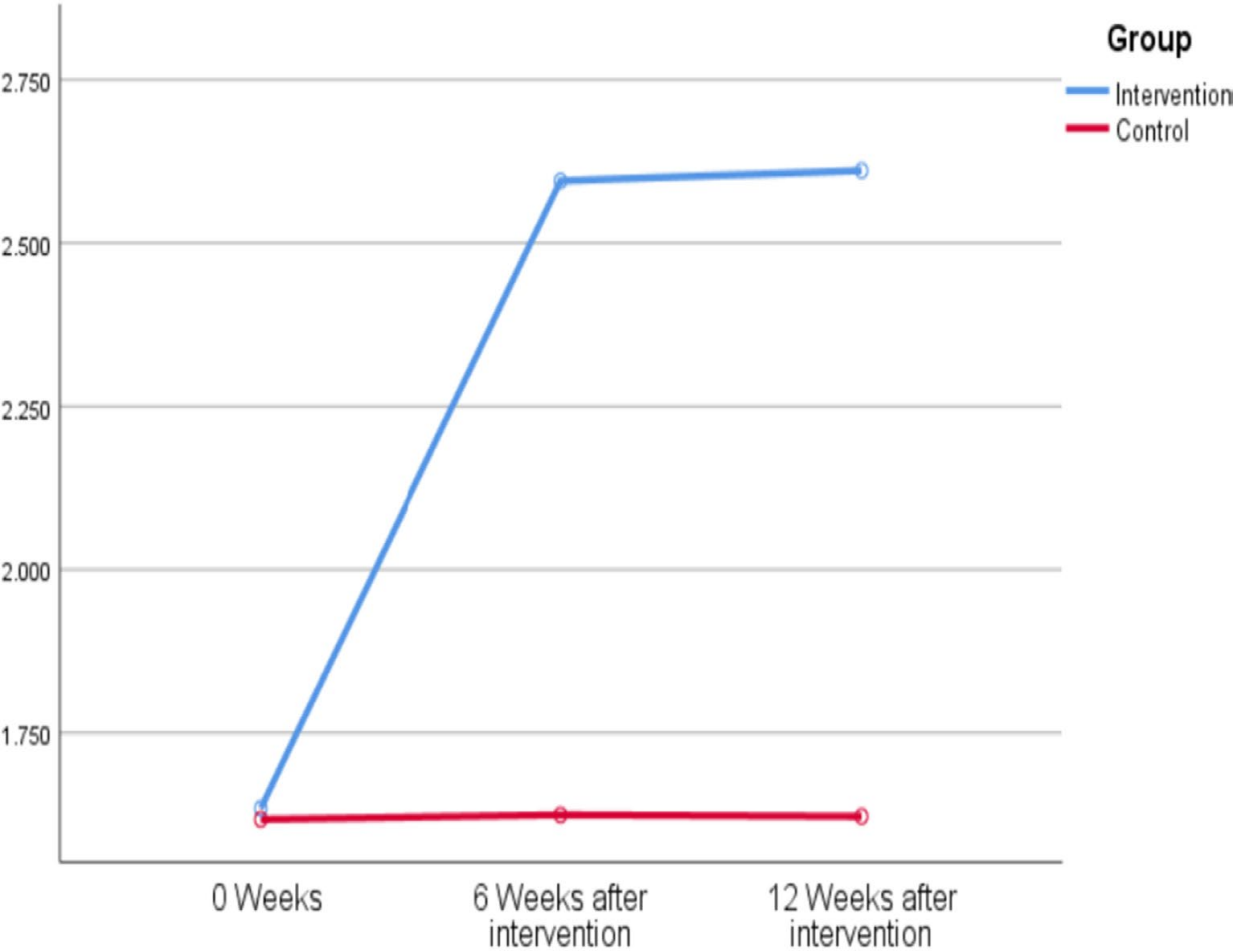
Comparison of mean scores knowledge.

**FIGURE 3:**
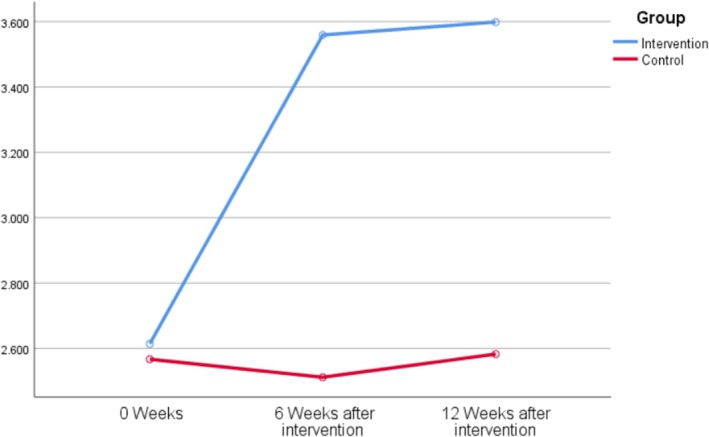
Comparison of mean scores attitude.

**FIGURE 4:**
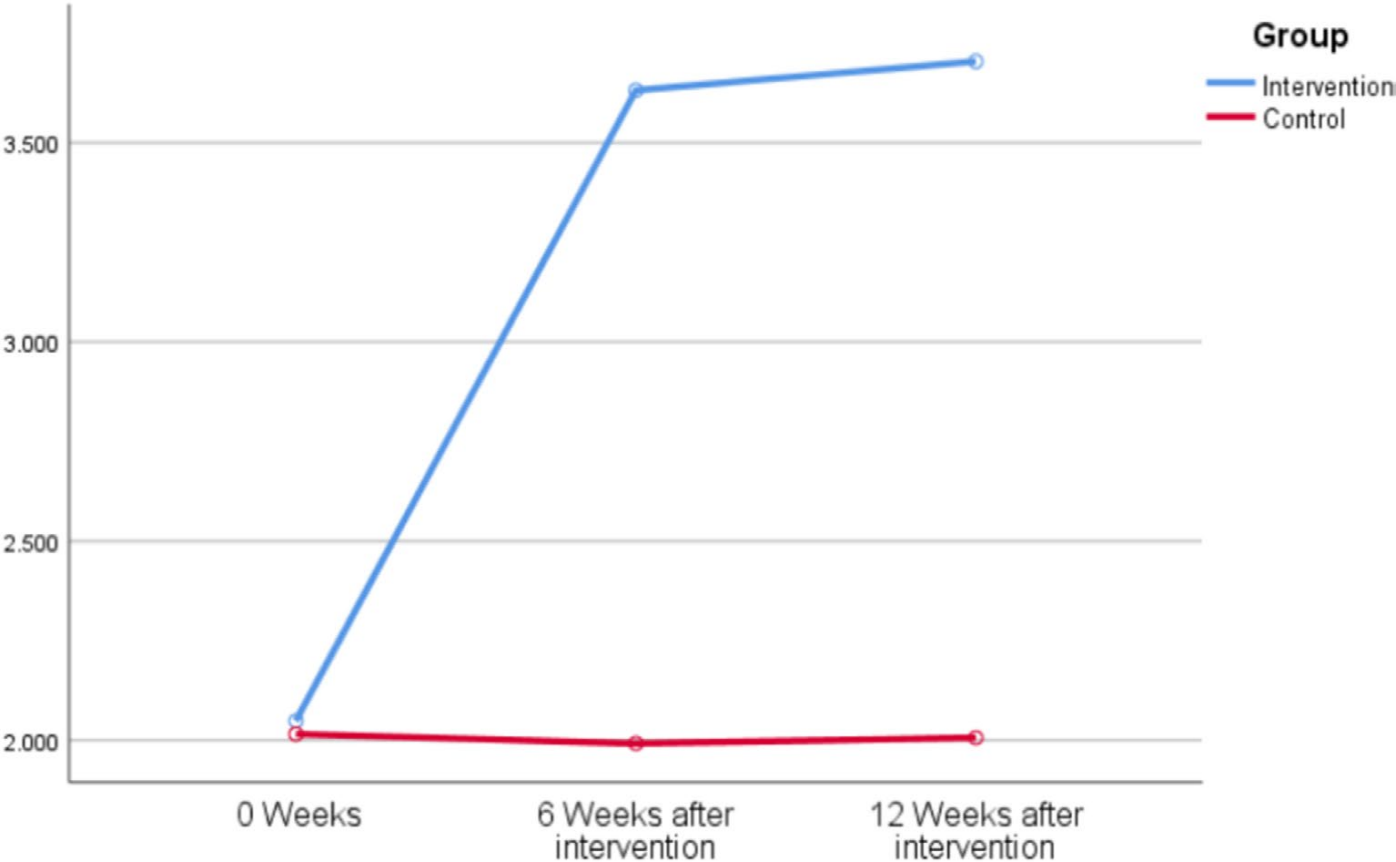
Comparison of mean scores self‐efficacy.

**FIGURE 5:**
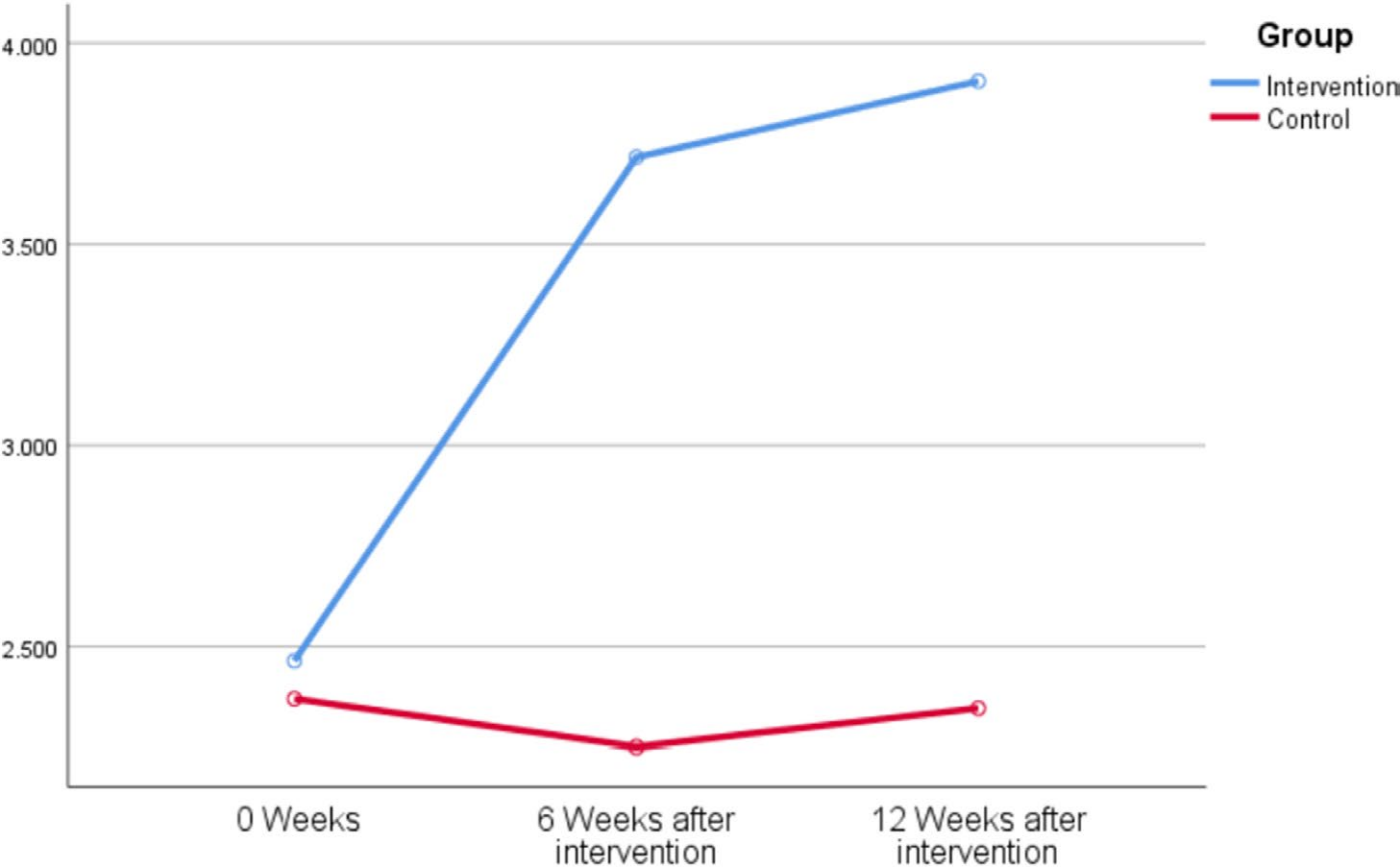
Comparison of mean scores reinforcing factors.

**FIGURE 6:**
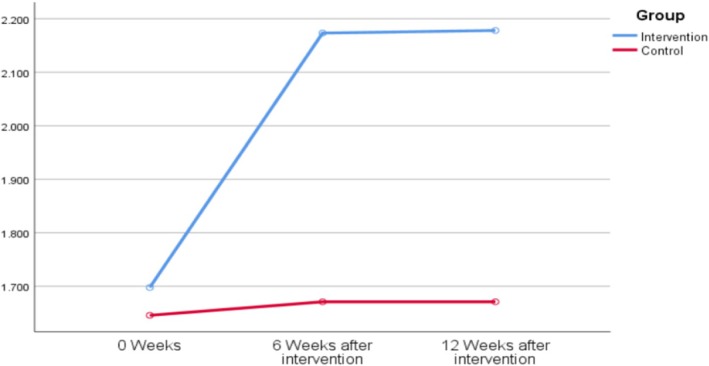
Comparison of mean scores enabling factors.

**FIGURE 7:**
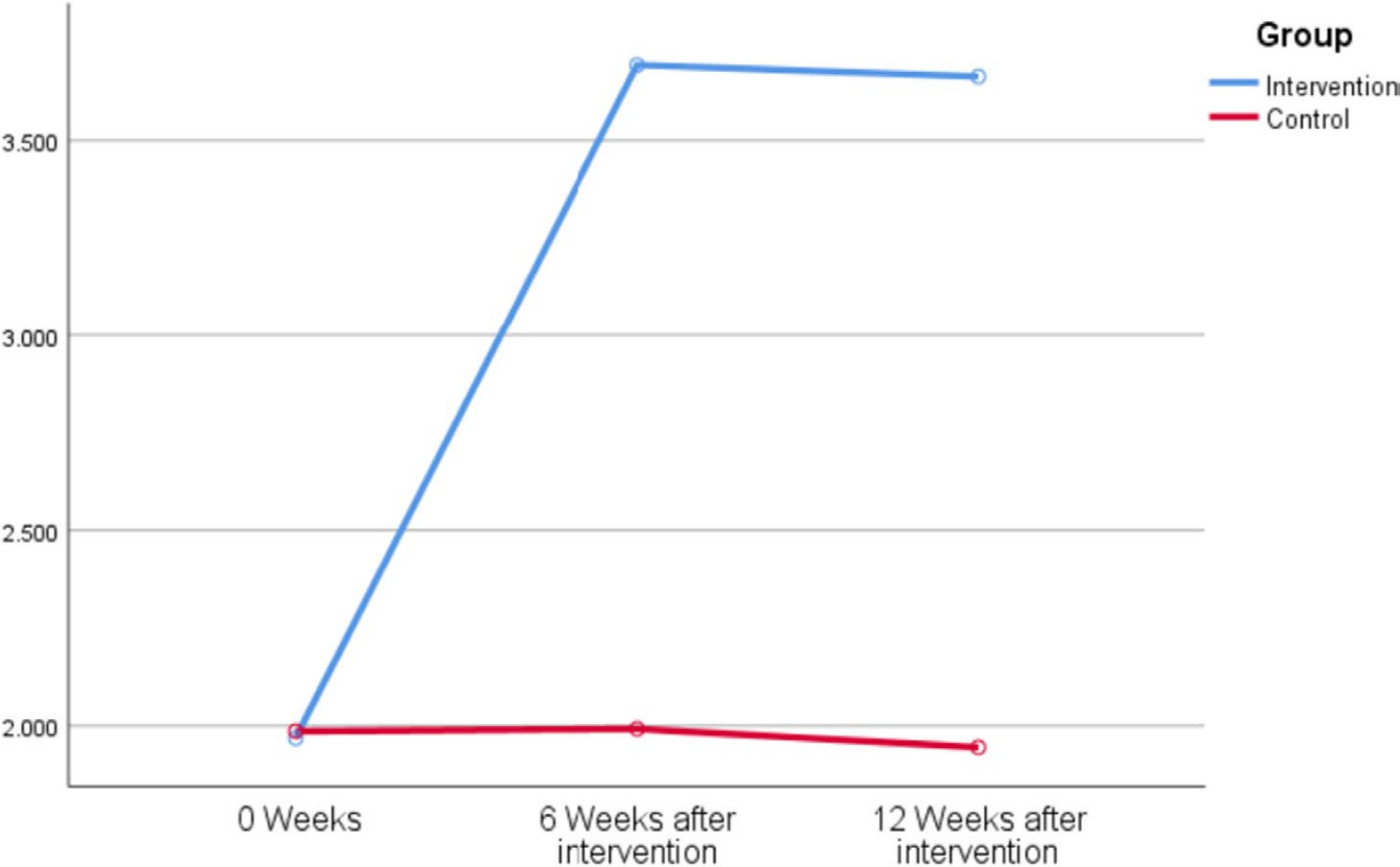
Comparison of mean scores behavior.

**FIGURE 8:**
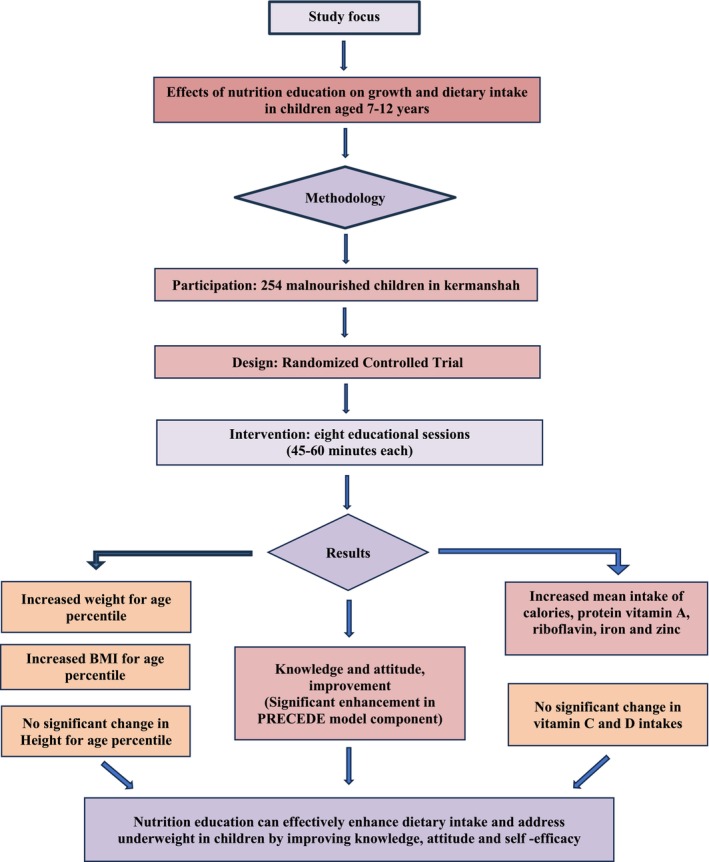
Study Summary Flowchart.

Table 3 presents the changes in the scores of the PRECEDE‐ PROCEED Model subscales at the beginning and end of the study, as well as between the two groups at the end of the study. After analyzing the PRECEDE‐ PROCEED Model subscales, it was observed that the PRECEDE model scores, including the scores of predisposing factors (knowledge, attitude and self‐efficacy), reinforcing factors, enabling factors, and behavior, had a significant increase in the intervention group's scores at Weeks 4 and 12 after the implementation of the educational program compared to the control group (Figures 2, 3, 4, 5, 6). It can be observed that the educational program had the greatest impact on elevating the scores of the self‐efficacy and behavior scales, so that in the intervention group, 1.66 and 1.7 points of increase in the scores of these two scales were observed 12 weeks after the educational program (*p* < 0.001) (Figures 7 and 8; Table 3).

**TABLE 3:** Mean scores of the PRECEDE‐PROCEED model before and after intervention in the studied groups.

Variables	Intervention group (*N* = 127) Mean ± SD				Control group (*N* = 127) Mean ± SD				*p* *
	Week 0	Week 6	Week 12	*p* **	Week 0	Week 6	Week 12	*p* **	
Knowledge	1.63 ± 0.20	2.59 ± 0.19	2.61 ± 0.21	< 0.001	1.61 ± 0.18	1.62 ± 0.21	1.62 ± 0.18	0.955	< 0.001
Attitudes	2.61 ± 0.83	3.55 ± 0.59	3.59 ± 0.62	< 0.001	2.56 ± 0.80	2.51 ± 0.62	2.58 ± 0.70	0.652	< 0.001
Self‐efficacy	2.04 ± 0.59	3.63 ± 0.42	3.70 ± 0.37	< 0.001	2.01 ± 0.56	1.99 ± 0.56	2 ± 0.57	0.833	< 0.001
Reinforcing factors	2.46 ± 1.50	3.71 ± 0.88	3.90 ± 0.52	< 0.001	2.37 ± 1.50	2.25 ± 1.48	2.34 ± 1.49	0.129	< 0.001
Enabling factors	1.69 ± 0.24	2.17 ± 0.27	2.14 ± 0.28	< 0.001	1.64 ± 0.22	1.67 ± 0.26	1.66 ± 0.23	0.306	< 0.001
Behavior	1.96 ± 0.77	3.69 ± 0.47	3.66 ± 0.48	< 0.001	1.98 ± 0.76	1.99 ± 0.74	1.94 ± 0.68	0.483	< 0.001

*Note:* Data are presented as mean ± SD (standard deviation), CI, confidence interval. *p* value is significant at *p* < 0.05.

* *p* value represents significance/non‐significance of the resultant score for before and after two evaluations.

** *p* value is for comparison within control and intervention groups.

### 4.4 Food Intake

Food intake analysis was performed based on food consumption using a food record and analyzed using Nutritionist IV software. It indicated a significant rise in calories and macronutrient intake at Weeks 4 and 12 in the intervention group (*p* < 0.001). Also, 6 and 12 weeks after the implementation of the educational program, the average intake of B vitamins, vitamin A, beta‐carotene, copper, fiber, selenium, iron, zinc, calcium, magnesium, and phosphorus significantly increased in the intervention group (*p* < 0.001). However, no significant difference was observed in the average intake of vitamin C (*p* = 0.675) and vitamin D (*p* = 0.610) between the two groups 6 weeks after the intervention and 12 weeks after the intervention (Table 4).

**TABLE 4:** Dietary intake of subjects before and after intervention in the studied groups.

Variable	Intervention group				Control group				*p* *
	0 Week	6 Weeks	12 Weeks	*p*	0 Weeks	6 Weeks	12 Weeks	*p*	
Energy (kcal)	879 ± 104	1219 ± 158	1443 ± 162	< 0.001	890 ± 99.6	897 ± 104	912 ± 131	0.294	< 0.001
Carbohydrates (g)	140 ± 23	170 ± 40	198 ± 32	< 0.001	139 ± 24	142 ± 29	145 ± 28	0.171	< 0.001
Protein (g)	24 ± 10	44 ± 6	54 ± 7	< 0.001	23 ± 7	24 ± 10	23 ± 8	0.101	< 0.001
Fat (g)	25.52 ± 8	38.10 ± 15	54.39 ± 11	< 0.001	26.21 ± 8	25.09 ± 10	24.86 ± 11	0.466	< 0.001
VitB1 (mg)	0.65 ± 0.16	0.89 ± 0.14	0.91 ± 0.18	< 0.001	0.66 ± 0.14	0.65 ± 0.14	0.67 ± 0.13	0.66	< 0.001
VitB2 (mg)	0.55 ± 0.23	1.04 ± 0.25	1.09 ± 0.28	< 0.001	0.54 ± 0.12	0.53 ± 0.11	0.52 ± 0.10	0.709	< 0.001
VitB3 (mg)	7.67 ± 1.26	10.31 ± 1.57	10.61 ± 1.59	< 0.001	7.60 ± 1.24	7.70 ± 1.49	7.66 ± 1.34	0.353	< 0.001
VitB5 (mg)	2.04 ± 0.38	2.40 ± 0.38	2.54 ± 0.40	< 0.001	2.02 ± 0.32	2 ± 0.32	2.01 ± 0.31	0.513	< 0.001
VitB6 (mg)	0.68 ± 0.12	0.94 ± 0.14	1.04 ± 0.15	< 0.001	0.69 ± 0.10	0.69 ± 0.11	0.67 ± 0.08	0.175	< 0.001
VitB9 (mg)	129 ± 34	191 ± 33	212 ± 35	< 0.001	130 ± 35	132 ± 35	132 ± 34	0.471	< 0.001
VitB12 (mcg)	0.95 ± 0.27	1.56 ± 0.30	1.71 ± 0.24	< 0.001	0.96 ± 0.26	0.97 ± 0.29	0.96 ± 0.24	0.653	< 0.001
VitD (μg)	0.52 ± 0.37	0.54 ± 0.52	0.55 ± 0.57	0.913	0.52 ± 0.38	0.51 ± 0.35	0.53 ± 0.48	0.831	0.610
VitA (RE)	241 ± 82	401 ± 120	442 ± 114	< 0.001	238 ± 89	236 ± 88	235 ± 77	0.480	< 0.001
VitC (mg)	53.10 ± 34.6	55.31 ± 29.5	57.43 ± 27.9	0.296	53.86 ± 23.08	54.56 ± 22.54	54.09 ± 21.9	0.716	0.675
Betacarot (μg)	541 ± 227	793 ± 280	956 ± 117	< 0.001	555 ± 279	561 ± 274	552 ± 248	0.362	< 0.001
Ca (mg)	283 ± 116	647 ± 181	694 ± 168	< 0.001	294 ± 110	309 ± 138	304 ± 154	0.363	< 0.001
Fe (mg)	6.17 ± 1.31	9.44 ± 1.42	10.71 ± 1.53	< 0.001	6 ± 1.21	6.09 ± 1.20	6.04 ± 1.26	0.423	< 0.001
Zn (mg)	3.79 ± 1.94	6.60 ± 2	7.54 ± 1.63	< 0.001	3.66 ± 1.88	3.64 ± 1.71	3.59 ± 1.63	0.664	< 0.001
Cu (mg)	0.73 ± 0.22	0.93 ± 0.21	1.10 ± 0.19	< 0.001	0.72 ± 0.23	0.72 ± 0.22	0.74 ± 0.25	0.268	< 0.001
Fiber (g)	7.83 ± 1.69	11.39 ± 2.11	12.78 ± 2.05	< 0.001	7.78 ± 1.81	7.97 ± 1.82	7.80 ± 1.70	0.116	< 0.001
Se (mg)	0.026 ± 0.01	0.062 ± 0.07	0.064 ± 0.01	< 0.001	0.027 ± 0.02	0.028 ± 0.03	0.027 ± 0.01	0.968	< 0.001
Mg (mg)	181 ± 50	295 ± 77	300 ± 87	< 0.001	182 ± 60	182 ± 79	182 ± 70	0.996	< 0.001
P (mg)	374 ± 117	610 ± 164	738 ± 150	< 0.001	373 ± 125	375 ± 128	374 ± 114	0.948	< 0.001

* *p* values are a result of One‐Way Repeated Measures ANOVA test.

## 5 Discussion

Given the deficit of macronutrient and micronutrient intake, as well as the prevalence of malnutrition and its impact on the development of chronic diseases in adulthood, several studies have suggested immediate intervention in this area (Jafari Nodoushan et al. 2021; Suliga et al. 2018). Many studies have been conducted based on nutritional supplements, but there is insufficient information about the impact of nutritional education on growth status as macronutrient and micronutrient intake in malnourished children. One review study has summarized the findings of 94 separate studies and found largely low‐certainty evidence to suggest that dietary advice given with or without oral nutritional supplements may improve nutritional intake, weight, and quality of life in some adults with disease‐related malnutrition or at nutritional risk. The results were inconsistent and there were no clear trends related to which intervention might be the most beneficial or the length of time needed for the intervention to be effective (Baldwin et al. 2021). Findings of studies in children focused on reformulating dietary supplements, the importance of various health, dietary, and sociodemographic factors in influencing decisions regarding dietary supplementation in children. Studies also emphasized raising awareness among families about the use of these products when necessary and with the recommendation of a physician (Koç et al. 2024; Piorecka et al. 2024; Bailey et al. 2012).

Since one of the main reasons for malnutrition in children is inadequate health and nutritional information, providing correct information through nutritional education to children and their parents seems essential. Anthropometric indices are one of the tools that reflect the nutritional status in children. Based on the findings of the current study, we observed a significant relationship in WFA and BFA at 6 and 12 weeks after the intervention between the two groups, which indicates the effect of the nutrition education intervention on improving growth status (Table 2). Several studies conducted in infants and children have examined the effect of nutritional education on improving children's weight and height growth (Hu et al. 2010; Shenavar et al. 2022). The Shenavar study showed a positive effect of maternal nutritional education in the area of complementary feeding on improving WFA in children aged 6–59 months (Shenavar et al. 2022). Also, the Gumelar study, which examined the relationship between maternal nutritional education on local food intake and the anthropometric index of children aged 6–21 months, observed a significant difference in WFA (Gumelar and Tangpukdee 2022). The Walsh study inspected parental nutritional education of children aged 2–5 years, which observed a significant difference in WFA in urban areas (Walsh et al. 2002). It seems that the increase in WFA and body mass index for age was due to the increase in mothers' awareness of food sources rich in protein, iron, zinc, vitamin A, and riboflavin and their use in meals and snacks (Castro et al. 2017; Rerksuppaphol and Rerksuppaphol 2016; Sarma et al. 2006; Sivakumar et al. 2006). Numerous studies have shown the effect of nutrients on linear growth and the activity as well as increase in the secretion of hormones such as growth hormone (GH), IGF‐1, thyroid hormones, and glucocorticoids. These hormones are involved in the growth process by affecting skeletal muscles and bone plates (Millward 2017; Palacios 2006). Also, micronutrients such as selenium, iron, zinc, and vitamin A boost the secretion of thyroid hormone, which stimulates bone formation by regulating chondrocytes and cartilage matrix synthesis directly or in cooperation with GH. Vitamin A also modulates the GH gene through the RXR‐α receptor (Hesketh and Villette 2006; Inzaghi et al. 2022). Micronutrients such as zinc and selenium are required for protein synthesis and cell division. On the other hand, the increase in protein and amino acids stimulates GH plus IGF‐1 hormone, and hence skeletal muscle synthesis. Leucine is also an amino acid that is found in milk and some cereals which increases insulin through the MTOR pathway and elevates the hormone IGF‐1, which stimulates the maturation and differentiation of cartilage cells (Inzaghi et al. 2022; Hasan et al. 2024; Palmer et al. 2024; Trzeciakiewicz et al. 2009). The conclusion of the studies on the mechanisms mentioned is that improving nutritional status and enhancing the consumption of food sources rich in zinc, vitamin A, calcium, and iron can improve growth in children with growth disorders.

Studying children's growth further, no significant difference was observed between the two groups in HFA. Since height growth is a physiological phenomenon that takes time and does not occur quickly as with weight, and especially the height growth of children within the age range of 7–12 years is not significant, it seems logical that there is no significant change in height growth during the 20 weeks of the present study. The effect of any nutritional intervention on height growth does not occur in the short term, and studies have always recommended the continuation of effective interventions to observe improvements in height growth in children (Gumelar and Tangpukdee 2022; Salehi et al. 2008). In the present study, the mean scores of preventive behaviors for malnutrition in the intervention group rose significantly compared to the control group. Consuming breakfast, using healthy snacks at school, and reducing snack consumption could be due to the effect of nutritional education on awareness, attitude, self‐efficacy, as well as reinforcing and enabling factors. Another study conducted by Kashfi revealed that education based on the Ask model improved mothers' behavior in preventing growth disorders in children aged 6–12 months (Kashfi et al. 2014).

In the present study, a significant difference was observed in the total PRECEDE‐PROCEED Model scores between the two groups 6 and 12 weeks after the intervention; in particular, the scores of predisposing factors including knowledge, attitude, and self‐efficacy were significantly higher in the intervention group. One way to raise awareness is to provide nutritional education, which leads to the establishment of good eating habits. In addition, changing attitudes and increasing self‐efficacy led to a positive attitude toward breakfast consumption, a tendency to increase milk, vegetable, and meat consumption, and the use of nutritional recommendations to create appropriate nutritional behaviors. Increased self‐efficacy and attitude directly affect behavior change and its improvement (Azar et al. 2017; Fathi et al. 2016). In the present study, nutritional education based on the PRECEDE model led to behavioral changes in food choices in children and parents. Since in the studied age group, both children and parents have been influential in children's food choices, the necessary education was provided to both groups to enable changes in nutritional behaviors and acceptance of the newly taught foods. The findings of the present study indicated that there was a significant difference between the two groups in the average intake of calories, protein, fat, carbohydrates, B vitamins, vitamin A and beta‐carotene, copper, fiber, selenium, iron, zinc, calcium, magnesium, and phosphorus. Since education based on the PRECEDE‐PROCEED Model leads to healthy eating behaviors in children (Arshad et al. 2023), it seems that this increase in average food intake in the intervention group is due to enhanced awareness of children and their parents about food sources containing protein, vitamins, and minerals essential for growth, as well as positive changes in attitude and behavior in children. In the educational sessions of the present study, a sample meal and snack for children's growth was presented to parents to further familiarize them with the concept of growth‐promoting foods. Also, cost‐effective food substitutes, especially for protein‐rich food sources that often account for a high cost in the household food basket, were introduced to parents and children, which had a significant impact on improving the intake of macronutrients and micronutrients such as iron and zinc.

Similar studies have examined the effect of nutrition education on improving the nutrient intake of children and adolescents and have observed contradictory results (Antwi et al. 2020; Guo et al. 2013; Khani Jeihooni et al. 2023; Lee et al. 2009; Mushaphi et al. 2015). One reason for the contradictory results may be the difference in the duration of the study. Education is a learning‐based experience aimed at creating gradual and relatively permanent changes in an individual to improve their ability to perform tasks. Since education is a continuous activity, it can induce changes in skills, knowledge, attitudes, and behavior when a sufficient period of time is allocated to it. Meanwhile, one of the important issues in changing eating habits is the introduction of affordable food alternatives. Since most protein‐rich sources are expensive, it may not be possible to include them in the food basket despite awareness and knowledge of the importance of consuming these foods, which will especially harm children in a family. In addition, protein‐energy malnutrition is highly prevalent in marginal areas. Thus, teaching affordable and economical alternatives, which was one of the most fundamental aspects of education in the present study, could be the main key to achieving nutritional goals in improving children's growth (Kim and Kim 2012; Li et al. 2022).

Regarding the PRECEDE‐PROCEED Model subscales, the present study found an improvement in reinforcing and enabling factors in the intervention group, which could indicate that parents and teachers encouraged children to change their lifestyles and increased their willingness to make positive changes in behavior. It also seems that the use of educational resources introduced by educators to malnourished children and their parents improved their self‐efficacy. Other studies that used this educational model to improve self‐care in patients also found similar results (Azar et al. 2017; Fadaei et al. 2021; Fitriani et al. 2020).

According to the results of our study, nutrition education based on the PRECEDE‐PROCEED Model can be a useful and low‐cost tool to improve the malnutrition status of children. The strengths of our study have been the repeated assessment of participants and the use of the PRECEDE‐PROCEED Model, which is one of the most common health education models and includes multilevel planning and evaluation, which allows for greater efficiency. Our study had the limitation of examining physical activity in children, so the findings should be interpreted with caution.

## 6 Conclusion

The findings of our study revealed that nutrition education based on the PRECEDE‐PROCEED Model can increase awareness, attitude, self‐efficacy, and encourage parents and teachers to create positive nutritional behaviors as well as improve food intake in school‐age children, thereby reducing the incidence of underweight and wasting in them. According to the results of this study, it is recommended that nutrition education content be included in school curricula and that nutritionists widely teach and develop healthy eating plans in schools.

## Author Contributions

All authors made substantial contributions to conception and design, acquired data, drafted the article or revised it critically for important intellectual content; gave final approval of the version to be published; and agreed to be accountable for all aspects of the work.

## Ethics Statement

The protocol was approved by the Medical University of Kermanshah Research Ethics Board (IR.KUMS.REC.1402.137). Also, a written consent form will be obtained from the children's parents as well as verbal consent from the children.

## Conflicts of Interest

The authors declare no conflicts of interest.

## Data Availability

The data that support the findings of this study are available from the corresponding author, upon reasonable request.
